# Dietary ω-3 Polyunsaturated Fatty Acids Inhibit Tumor Growth in Transgenic Apc^Min/+^ Mice, Correlating with CB1 Receptor Up-Regulation

**DOI:** 10.3390/ijms18030485

**Published:** 2017-02-24

**Authors:** Maria Notarnicola, Valeria Tutino, Valentina De Nunzio, Francesco Dituri, Maria Gabriella Caruso, Gianluigi Giannelli

**Affiliations:** National Institute of Gastroenterology “S. de Bellis”, Research Hospital, Castellana Grotte, Bari 70013, Italy; maria.notarnicola@irccsdebellis.it (M.N.); valeria.tutino@irccsdebellis.it (V.T.); valentinadx@hotmail.it (V.D.N.); francesco.dituri@irccsdebellis.it (F.D.); gabriella.caruso@irccsdebellis.it (M.G.C.)

**Keywords:** olive oil, ω-3 polyunsaturated fatty acids, CB1 receptor, colon cancer, transgenic Apc^Min/+^ mice

## Abstract

Mediterranean diet components, such as olive oil and ω-3 polyunsaturated fatty acids (ω-3 PUFAs), can arrest cell growth and promote cell apoptosis. Recently, olive oil has been demonstrated to modulate type-1 cannabinoid (*CB1*) receptor gene expression in both human colon cancer cells and rat colon. The aim of this study was to investigate a possible link between olive oil and ω-3 PUFAs effects and CB1 receptor expression in both intestinal and adipose tissue of Apc^Min/+^ mice. To confirm the role for the CB1 receptor as a negative modulator of cell proliferation in human colon cancer, *CB1* receptor gene expression was also detected in tumor tissue and in surrounding normal mucosa of patients with colorectal cancer (CRC). Dietary ω-3 PUFAs significantly inhibited intestinal polyp growth in mice, correlating with *CB1* receptor gene and protein expression induction. *CB1* receptor gene up-regulation was also detected in adipose tissue, suggesting a close communication between cancer cells and the surrounding environment. Tissue CB1 receptor induction was associated with a concurrent inactivation of the Wnt/β-catenin pathway. Moreover, there was a significant reduction in *CB1* receptor gene expression levels in cancer tissue compared to normal surrounding mucosa of patients with CRC, confirming that in cancer the “protective” action of the CB1 receptor is lost.

## 1. Introduction

The evidence for the health benefits of dietary ω-3 polyunsaturated fatty acids (ω-3 PUFAs) has been drawn from different epidemiologic and controlled intervention studies [1,2]. PUFAs can inhibit colon carcinogenesis, affecting tumor progression [3]. Previously, we showed the ability of ω-3 PUFAs to arrest cell growth and to promote cell apoptosis in animal and in vitro studies [4,5,6]. Moreover, ω-3 PUFAs ingested in the diet were also able to significantly reverse polyp development in Apc^Min/+^ mice through inducing the estrogen receptor β and Low Density Lipoprotein (LDL) receptor [7], known to be negative modulators of cell proliferation.

Several molecular pathways influencing cell proliferation activities have been identified. Among them, the endocannabinoid system (ECS) plays an important role [8]. Cannabinoids receptor agonists have been demonstrated to have an antitumor action, mostly via Cannabinoid receptor-1 (CB1) activation. We previously demonstrated that anandamide (Met-F-AEA), an endogenous agonist for the CB1 receptor, inhibits the proliferation of colon cancer cell lines. This anti proliferation effect was related to a significant reduction of cells in the S phase of the cell cycle and to a decrease of the cell polyamines content [9]. We have also demonstrated an estrogenic induction of the CB1 receptor at mRNA and protein level in human colon cancer cells, suggesting a possible role of the CB1 receptor and its ligands as cell proliferation mediators [10].

CB1 receptor expression seems to be modulated by bioactive food components [11,12]; quercetin, the main member of the flavonoids, exerts anti-proliferative and pro-apoptotic effects in colon cancer cell lines, via CB1 receptor induction [11]. In addition, a significant up-regulation of *CB1* receptor gene has been observed in Caco2 cells after olive oil exposure [12]. *CB1* receptor gene modulation by olive oil takes place through epigenetic mechanisms, partly associated to a reduction in DNA methylation at gene promoter level [12].

Aim of this study was to investigate a possible link between olive oil and ω-3 PUFAs effects and *CB1* receptor gene and protein expression in mice that spontaneously develop intestinal polyps (Apc^Min/+^ mice).

In order to study the complex interactions between cancer cells and the tumor microenvironment, we evaluated *CB1* receptor gene expression levels in both intestinal and adipose tissue of these mice. In addition, to confirm the role for the CB1 receptor as a negative modulator of cell proliferation in human colon cancer, *CB1* receptor gene expression was also detected in tumor tissue and in surrounding normal mucosa of patients with colorectal cancer (CRC).

## 2. Results

**Figure 1:**
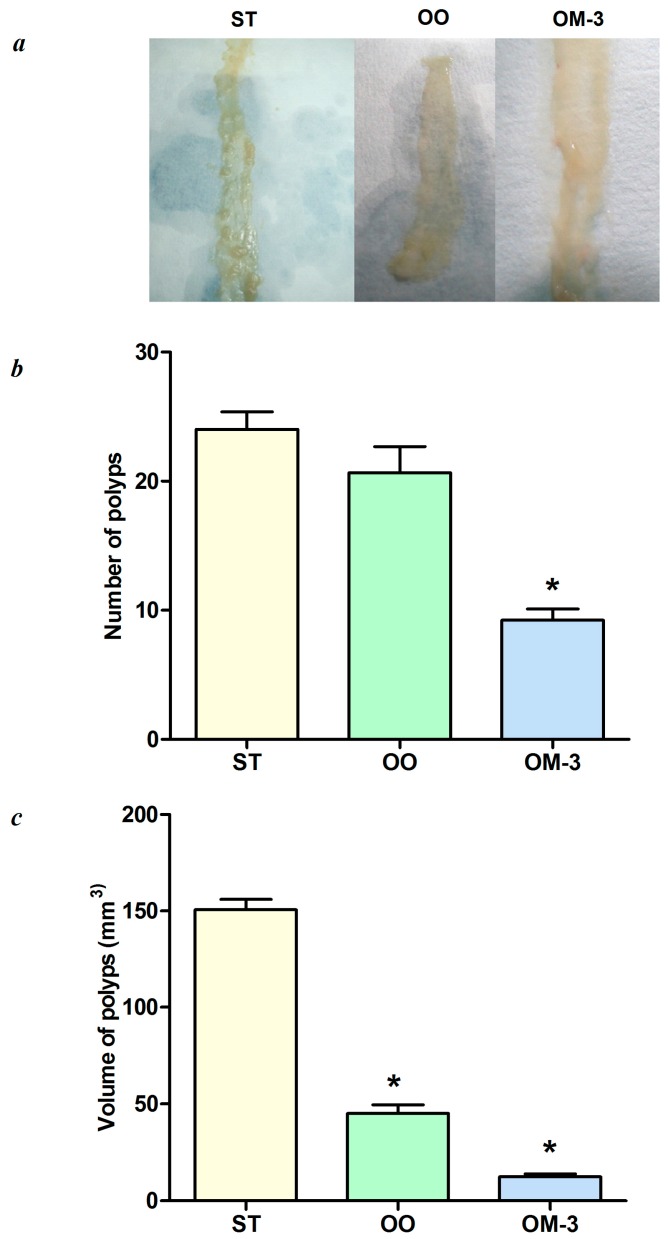
(**a**) Macroscopic view of intestinal tumors in the groups of mice that received three different dietary treatments; ST = standard diet; OO = standard diet with 12% olive oil; OM-3 = standard diet with 12% of salmon fish with a rich content of ω-3 PUFAs; (**b**) total number of intestinal polyps in the three mice treatment groups; (**c**) total volume of intestinal polyps in the three mice treatment groups. Data are presented as the mean ± SE. * *p* < 0.05 (ANOVA and Dunnett’s multiple comparison test, each experimental group vs. the ST group).

Figure 1, panel a shows intestinal samples cut along the mesenteric insertion, placed on a paper strip and analyzed. After 10 weeks of dietary treatment, there were macroscopic differences in polyp number and volume among the three mice groups. In accordance with our previous studies, the number and volume of polyps (Figure 1b,c, respectively) in mice treated with olive oil and ω-3 PUFAs were significantly decreased as compared to polyps detected in mice fed standard diet (*p* < 0.05, ANOVA and Dunnett’s multiple comparison test).

**Figure 2:**
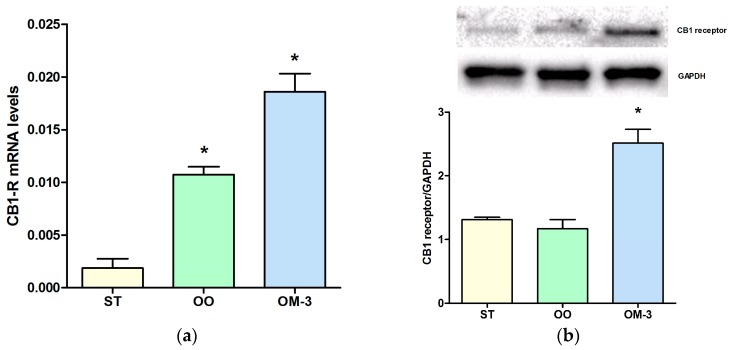
Cannabinoid receptor-1 (*CB1*) receptor gene expression levels (mean ± SE) in intestinal tissue of 10 Apc^Min/+^ mice for each group (**a**). Data expressed as molecule numbers of *CB1* receptor gene mRNA/molecule numbers of *β-actin* mRNA (**b**) Western blotting analysis of expression of the CB1 receptor protein in intestinal tissue of Apc^Min/+^ mice. ST = control group; OO = olive oil group; OM-3 = ω-3 PUFAs group. * *p* < 0.05 (ANOVA and Dunnett’s multiple comparison test, each experimental group vs. the control (ST) group).

**Figure 3:**
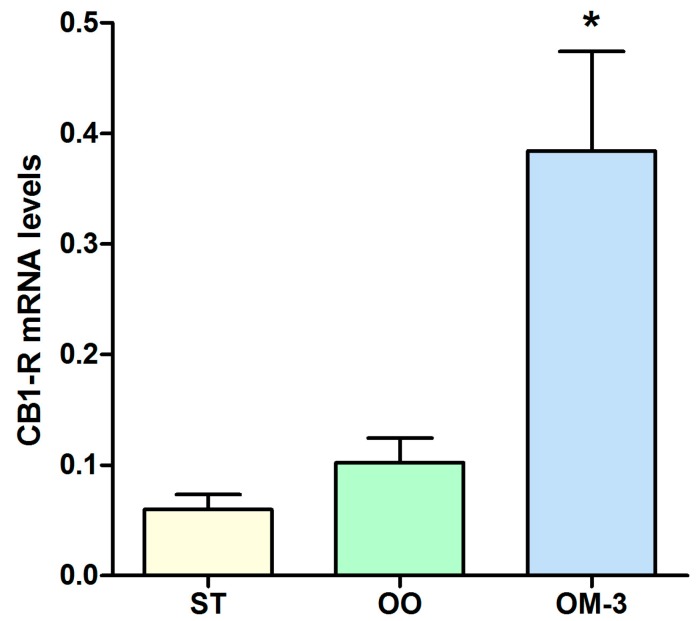
*CB1* receptor gene expression levels (mean ± SE) in adipose tissue of 10 Apc^Min/+^ mice for each group. Data expressed as molecule numbers of *CB1* receptor gene mRNA/molecule numbers of *β-actin* mRNA. * *p* < 0.05 (ANOVA and Dunnett’s multiple comparison test, each experimental group vs. the ST group).

**Figure 4:**
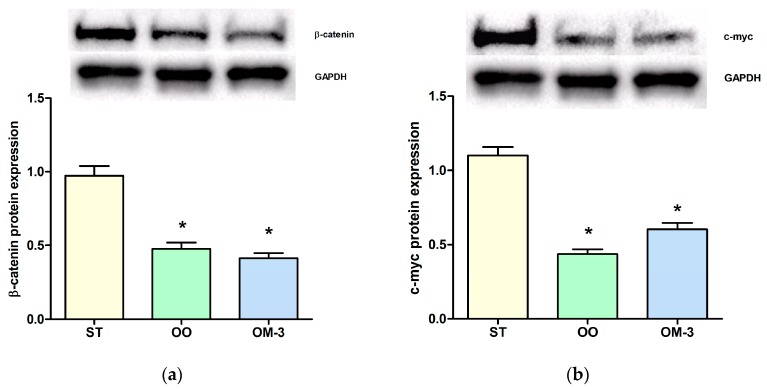
(**a**) western blotting analysis of expression of the β-catenin protein in intestinal tissue of treated Apc^Min/+^ mice; (**b**) western blotting analysis of expression of the c-myc protein in intestinal tissue of mice. ST = control group; OO = olive oil group; OM-3 = ω-3 PUFAs group. * *p* < 0.05 (ANOVA and Dunnett’s multiple comparison test, each experimental group vs. the ST group).

The anti-proliferative action of diets enriched with olive oil and ω-3 was correlated with an induction of *CB1* gene receptor expression. Analysis of *CB1* receptor gene expression levels in intestinal tissue revealed a statistically significant induction in the olive oil (OO) and ω-3 PUFAs (OM-3) group as compared to animals fed standard diet (*p* = 0.003 and *p* = 0.001, respectively, ANOVA and Dunnett’s multiple comparison test) (Figure 2a). Higher levels of CB1 receptor protein expression, in intestinal tissue, were detected in the OM-3 group compared to animals fed standard diet (Figure 2b, *p* = 0.002, ANOVA and Dunnett’s multiple comparison test), whereas no difference was found between the standard (ST) and OO groups. In adipose tissue, compared to the ST group, a striking increase of *CB1* receptor gene expression was detected in mice treated with ω-3 PUFAs (*p* = 0.001, ANOVA and Dunnett’s multiple comparison test), whereas a slight induction was detected in the OO group mice (Figure 3). In order to demonstrate a direct action of ω-3 PUFAs on some factor related to the β-catenin and c-myc context, we investigated the protein levels of β-catenin and c-myc, downstream effectors of CB1 receptor activity. Olive oil and ω-3 PUFAs treatment induced a significant reduction of β-catenin and c-myc protein expression (Figure 4a,b).

**Figure 5:**
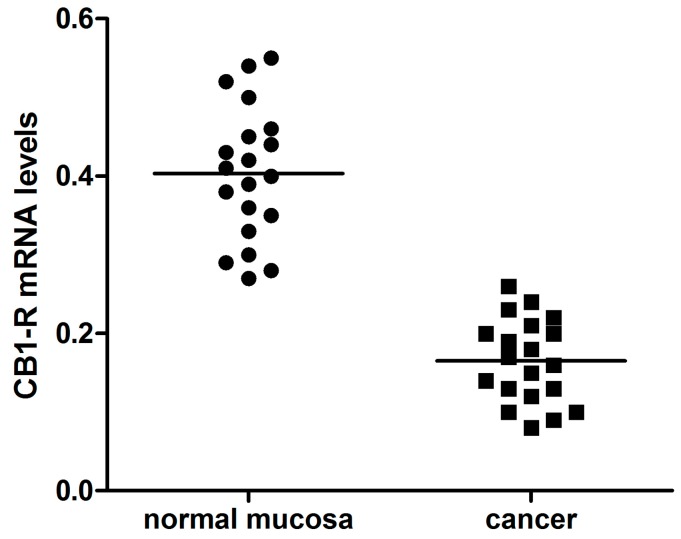
*CB1* receptor gene expression levels (mean ± SE) in intestinal normal mucosa and cancer tissue from 20 patients with colorectal cancer. Data expressed as molecule numbers of *CB1* receptor gene mRNA /molecule numbers of *β-actin* mRNA.

Confirming the CB1 receptor role in counteracting tumor progression, the levels of *CB1* receptor gene expression were significantly lower in cancer tissue compared to normal surrounding mucosa of patients with CRC (Figure 5, *p* = 0.01, paired *t*-test). No association was found between *CB1* receptor mRNA levels and age, sex, tumor site, disease stage and histological differentiation.

## 3. Discussion

The present study confirms the central role for natural compounds, such as olive oil and ω-3 PUFAs, in the regulation of cell proliferation. Two tested diets significantly inhibited intestinal polyp growth in Apc^Min/+^ mice, but dietary ω-3 PUFAs were able to control cell proliferation more efficaciously than olive oil. Moreover, we detected a correlation between the tumor suppressant effects of ω-3 PUFAs and the CB1 receptor up-regulation in these mice.

In association with the CB1 receptor up-regulation, we detected a reduced expression of β-catenin and its transcriptional target c-myc, both important players involved in cell proliferation.

Experimental evidence has already shown that the CB1 receptor is able to control cell proliferation through the Wnt/β-catenin signaling pathway cascade [11,13]. The Wnt signaling pathway is known to have a critical role in colorectal carcinogenesis [14]. Wnt/β-catenin is activated in approximately 90% of colorectal cancers [15] and its activity depends on the amount of β-catenin located in the cytoplasm. Our present data suggest that CB1 receptor induction promotes β-catenin degradation, antagonizing the canonical Wnt pathway.

Tumor growth is strongly affected by the complex interactions between cancer cells and the surrounding microenvironment [16]. Diet-derived compounds can contribute to control the cancer cells metabolism, affecting signaling events that take place in their neighboring cells. The CB1 receptor induction detected in both intestinal and adipose tissue from Apc^Min/+^ mice underlines this cross-talk between the tumor and surrounding environment. Nutritional intervention in Apc^Min/+^ mice counteracts intestinal carcinogenesis, improving the environmental conditions where tumors can develop.

We have previously demonstrated that adipose and colon tissue interact, affecting the enzymatic activity of proteins involved in lipogenesis and cell proliferation [17]. Moreover, pronounced morphologic and molecular alterations of adipose tissue have been observed in tumor-bearing mice [18]. Several studies have found a trophic effect of adipose tissue on colon cancer cell growth [17,19,20,21].

The CB1 receptor is considered as a tumor suppressor gene that exerts anti-proliferative effects on cancer cells [13,22]. CB1 receptor down-regulation has been correlated with a number of neurodegenerative diseases [23] and with different processes that participate in cancer development and likely influence its progression [24].

Consistent with the hypothesis that the *CB1* receptor gene acts as a negative modulator of cell proliferation, we detected a significant reduction of *CB1* receptor gene expression in cancer compared to normal surrounding mucosa from patients with CRC.

Several studies have reported a lack of the “protective” action of CB1 receptor in different types of cancer [25,26,27], indicating that absence or down regulation of the CB1 receptor frees neoplastic cells from mechanisms controlling cell growth and proliferation. 

In conclusion, we provide in vivo and ex vivo evidence that CB1 receptor induction is correlated with an inhibition of cell proliferation. In future studies, the use of CB1 receptor agonists and/or antagonists is warranted to ascertain whether the anti-proliferative effects of ω-3 PUFAs on intestinal polyp in Apc^Min/+^ mice are mediated by CB1 receptor up-regulation.

## 4. Material and Methods

### 4.1. Animals and Experimental Study Design

In vivo experiments were carried out as previously described [4]. Briefly, Apc^Min/+^ transgenic mice (five-weeks-old), were purchased from Charles River (Calco, CO, Italy) and maintained in controlled conditions and with a free access to food and water. The procedures related to animal use have been approved by the Italian Ministry of Health (N°103/2016) and conducted in adherence with the International Guidelines for the use of laboratory animals. Apc^Min/+^ mice were randomly divided into 3 groups of 10 animals each and fed for 10 weeks as follows: the control group (ST) received a standard diet (12.5% protein, 12% soya bean oil, 3% fiber); the OO group received a standard diet in which soya bean oil was replaced by olive oil (12.5% protein, 12% olive oil, 3% fiber); OM-3 group received a standard diet in which soya bean oil was replaced by 12% of salmon fish with a rich ω-3 PUFAs content.

All diets were provided in pellet form (Mucedola srl, Settimo Milanese, Italy) and immediately stored at −80 °C to prevent PUFAs oxidation. Mice were given fresh food daily, and body weights were recorded weekly.

Animals were treated for 10 weeks and sacrificed by cervical dislocation. Samples of intestinal and adipose tissue were immediately removed and stored at −80 °C until assayed. 

### 4.2. Patients

Twenty consecutive CRC patients (9 females and 11 males, mean age 73.1 ± 12.4 years) undergoing surgery of the colon were enrolled in the study. Colorectal normal mucosa and cancer tissue were obtained from each of them. Specimens were taken within 1 h after the surgical procedure and stored at −80 °C until assayed. Clinical and histopathological features of each patient were recorded (Table 1) and all patients gave informed consent to take part in the study. The study was approved by the Ethics Committee of our Institute (N°41/2016).

### 4.3. CB1 Receptor Gene Expression Analysis

*CB1* receptor mRNA levels, in intestinal and in adipose tissue of mice, as well as in colorectal tumor and in surrounding normal mucosa of patients with CRC, were analyzed by real-time RT-PCR assay. Total tissue RNA, isolated with TRI-Reagent (Mol. Res. Centre Inc., Cincinnati, OH, USA), was reverse transcripted in 20 μL of the final volume at 41 °C for 60 min, using 30 pmol antisense primer for *CB1* receptor and *β-actin* gene (Table 2). Real-time PCR was carried out as previously described [4]. All expression data were normalized by dividing the target amount by the amount of *β-actin*, used as internal control. Gel electrophoresis was used to confirm the specificity of PCR products.

### 4.4. Western Blotting

Protein samples were subjected to electrophoresis using SDS-PAGE gel and subsequently transferred onto a PVDF (polyvinylidene difluoride) membrane (Bio-Rad Laboratories, Milan, Italy) and probed with anti-β-catenin, anti-c-myc and anti-GAPDH primary antibodies (Cell Signaling, Beverly, MA, USA). After overnight incubation, a horseradish peroxidase-conjugated secondary antibody (Bio-Rad Laboratories) was used. After chemiluminescence and densitometric analysis, the signal of each protein was obtained using the Molecular Imager ChemidocTM (Bio-Rad Laboratories) and normalized against GAPDH expression.

### 4.5. Statistical Analysis

One-way analysis of variance (ANOVA) and Dunnett’s multiple comparison test were used to evaluate the significance of the differences among treated groups. The differences in CB1 receptor expression levels between normal mucosa and cancer and the association analysis with clinical parameters were detected by paired *t*-test and the χ^2^ test, respectively. Differences were considered statistically significant with a *p*-value < 0.05.

## Figures and Tables

**Table 1:** Clinical and histopathological features of patients.

Parameters		Patients (*n* = 20)
Age		73.1 ± 12.4 (mean ± SD)
Sex	Male	11
	Female	9
Tumor side ^1^ (R/L)	Right	8
	Left	12
Tumor stage ^2^	Stage I	2
	Stage II	8
	Stage III	7
	Stage IV	3
Histological grading	Well differentiated	2
	Moderately differentiated	12
	Poorly differentiated	6

^1^ Right side: hepatic flexure, cecum and ascending colon; left side: descending colon, sigmoid and rectum; ^2^ Clinical staging performed using the UICC (Union for International Cancer Control) system.

**Table 2:** Sequences of primers.

Gene	Primer	Sequence Primer
*Human CB1-R*	Sense	5′-GGAGAACATCCAGTGTGGGG-3′
	Antisense	5′-CATTGGGGCTGTCTTTACGG-3′
*Human β-actin*	Sense	5′-AAAGACCTGTACGCCAACACAGTGCTGTCTGG-3′
	Antisense	5′-CGTCATACTCCTGCTTGCTGATCCACATCTGC-3′
*Mouse CB1-R*	Sense	5′-CCTGGGCTGGAACTGCAA-3′
	Antisense	5′-CCGAAGACGTCATACACCATGA-3′
*Mouse β-actin*	Sense	5′-GCCTCTGGTCGTACCACTGGC-3′
	Antisense	5′-AGGGAGGAAGAGGATGCGGCA-3′
